# Relationship Between the Oral Microbiome and Treatment Efficacy in Esophageal Squamous Cell Carcinoma

**DOI:** 10.1245/s10434-025-18945-8

**Published:** 2026-01-12

**Authors:** Manato Ohsawa, Hiromi Nishi, Yoichi Hamai, Manabu Emi, Yuta Ibuki, Hitoshi Komatsuzawa, Hiroyuki Kawaguchi, Morihito Okada

**Affiliations:** 1 Department of Surgical Oncology, Hiroshima University, Hiroshima, Japan; 2 Department of General Dentistry, Hiroshima University Hospital, Hiroshima, Japan; 3 Department of Bacteriology, Hiroshima University Graduate School of Biomedical and Health Sciences, Hiroshima, Japan

**Keywords:** Oral microbiome, Diversity, Esophageal squamous cell carcinoma, Pathological response, Cancer treatment, Metagenomic analysis

## Abstract

**Background:**

As the relationship between oral microbiota and treatment efficacy in esophageal cancer remains unexplored, we aimed to clarify it using metagenomic analysis.

**Patients and Methods:**

Of the 140 consecutive patients with esophageal squamous cell carcinoma (ESCC) who underwent esophagectomy with R0 resection at Hiroshima University Hospital between April 2020 and May 2024, 74 who received neoadjuvant therapy were included in this study. 16S rRNA gene from oral tongue coating samples was amplified using polymerase chain reaction and subjected to next-generation sequencing. The oral microbiome data were analyzed using QIIME2 and linear discriminant analysis effect size, and the relationship between the oral microbiota and treatment efficacy and prognosis was assessed.

**Results:**

Alpha diversity of the oral microbiota was significantly correlated with the pathological response. Univariate and multivariate analyses showed that the alpha diversity of the oral microbiome (high versus low) was a significant predictor of a good pathological response. Patients with high alpha diversity had significantly improved recurrence-free survival and overall survival compared with those with low alpha diversity. Furthermore, eight bacterial groups (*Lactobacillales*, *Peptostreptococcales-Tissierellales*, *Bifidobacteriaceae*, *Erysipelotrichaceae*, *Lactobacillaceae*, *Anaerovoracaceae*, *Staphylococcaceae*, and *Aerococcaceae*) were significantly more abundant in individuals who responded well to neoadjuvant therapy and two bacterial groups (*Streptococcaceae* and *Corynebacteriaceae*) were significantly more abundant in poor responders.

**Conclusions:**

Our results demonstrate a correlation between the oral microbiome and ESCC treatment efficacy, suggesting that it is a significant prognostic factor. Our findings may also help predict the efficacy of esophageal cancer treatment.

**Supplementary Information:**

The online version contains supplementary material available at 10.1245/s10434-025-18945-8.

Esophageal cancer is the seventh most common type of cancer worldwide and the sixth most common cause of cancer-related deaths. Squamous cell carcinoma is the most common type of esophageal cancer, accounting for approximately 90% of all cases worldwide.^1^ Despite advances in multimodal therapies, such as surgery, chemotherapy, radiotherapy, and chemoradiotherapy, esophageal cancer remains a highly lethal malignancy, with a 5-year overall survival rate of 15–20%.^2^ Controlling locally advanced esophageal cancer and improving patient survival requires a trimodal strategy, i.e., a combination of neoadjuvant chemotherapy, radiotherapy, and surgery.^3,4^ Prognosis after trimodal therapy is related to the neoadjuvant therapy response of the patient.^5,6^ Therefore, accurately predicting treatment efficacy is crucial for guiding treatment decisions.

There is growing interest in the role of the gut microbiota in various malignancies, as evidence suggests that it may influence the development and progression of gastrointestinal cancers by regulation of gut inflammation and tumor-related signaling pathways.^7,8^ Further, the gut microbiota is involved in determining the therapeutic efficacy of chemotherapy and immunotherapy.^9,10^

Oral microbiota, including periodontal disease bacteria, is associated with various systemic conditions.^11,12^ Similar to the gut microbiota, the oral microbiota is associated with cancer; a positive correlation exists between oral bacteria and the risk of several types of cancer. Most reports have focused on its association with oral cancer and head and neck cancer, but associations with esophageal cancer have also been suggested.^13–15^

The advent of next-generation sequencing (NGS) in bacteriology has led to significant changes in methodology and analysis. For example, metagenomic analysis enables the comprehensive analysis of DNA from all microorganisms, thereby facilitating a deeper understanding of the microbiome’s characteristics. Oral bacterial analysis has been enhanced via meta-16S analysis, which specifically targets the 16S rRNA gene and subjects the same to next-generation DNA sequencing.^16^

Currently, only a few metagenomic data reports exist on the oral microbiota of patients with esophageal cancer, and no studies have investigated the relationship between oral microbiota and treatment efficacy. Here, we aimed to clarify the relationship between the oral microbiota of patients with esophageal cancer and the efficacy of neoadjuvant therapy using metagenomic analysis.

## Patients and Methods

### Patients

In total, 140 consecutive patients with esophageal squamous cell carcinoma (ESCC) underwent esophagectomy with R0 resection at Hiroshima University Hospital between April 2020 and May 2024. Of these, 74 patients who received neoadjuvant therapy were included in this study. The clinical data of the patients were obtained from surgical databases and medical records. The Institutional Review Board of Hiroshima University approved the study protocol (approval number: E-1874), and written informed consent was obtained from all patients.

### Clinical and Pathological Assessment

Tumor diagnosis was based on clinicopathological assessment conducted according to the tumor‐node‐metastasis classification (8^th^ edition).^17^ Clinical responses to neoadjuvant therapy and restaging examinations performed before surgery were evaluated according to the Response Evaluation Criteria in Solid Tumors (RECIST).^18^ The pathological response of the primary tumors was graded 0, 1, 2, and 3 according to the response evaluation criteria for the effects of radiation and/or chemotherapy published by the Japan Esophageal Society.^19^ Individuals with tumor grades 2 and 3 were defined as good responders and those with grades 0 and 1 as poor responders.

### Neoadjuvant Therapy

For neoadjuvant chemotherapy, cisplatin/5-fluorouracil (CF), nedaplatin/5-fluorouracil, and docetaxel/cisplatin/5-fluorouracil (DCF) regimens were administered to 16 (24.6%), 7 (10.8%), and 42 (64.6%) patients, respectively; administration schedules for CF and DCF regimens have been reported previously.^20^ For patients with impaired renal function who could not receive the CF regimen, cisplatin was replaced with nedaplatin.

Neoadjuvant chemotherapy was adopted as the initial neoadjuvant therapy; however, for patients with bulky primary tumors and obstructive symptoms, neoadjuvant chemoradiotherapy was used. Neoadjuvant chemoradiotherapy and surgery comprised concurrent radiotherapy (40 Gy in 20 fractions) and chemotherapy with 5-fluorouracil and cisplatin; radiation schedule and range have been described previously.^21^

### Surgical Treatment

Surgery was scheduled 3–5 weeks after the completion of neoadjuvant chemotherapy or 4–8 weeks after the completion of neoadjuvant chemoradiotherapy. All patients underwent open transthoracic (*n* = 9), thoracoscopic (*n* = 39), or robotic (*n* = 26) esophagectomy with lymph node dissection in at least two fields (thoracic and abdominal fields (*n* = 34). Esophageal cancer in the upper and middle thirds of the thoracic esophagus and lymph node metastases in the superior mediastinum was treated using cervical lymphadenectomy. A total of 40 patients underwent thoracic, abdominal, and cervical lymph node dissections. Subsequently, a gastric tube was inserted for cervical anastomosis of the esophagus. The reconstruction path was either retrosternal (*n* = 59), posterior to the mediastinum (*n* = 6), or located in the region before the chest wall (*n* = 9). The gastric tube was lifted for cervical anastomosis of the esophagus. Three experts in esophageal surgery performed the procedures.

### Sample Collection and NGS Library Preparation and Analysis

Before starting esophageal cancer treatment, a dentist at our hospital performed an oral examination and maintained oral hygiene. Sample collection was performed during the initial dental examination, and eating, drinking, and tooth brushing were avoided for at least 2 h before standardization. The tongue coating was collected by rubbing the dorsal surface of the tongue 10 times using a sterile sponge brush. DNA was extracted from the samples, and the hypervariable region V1–V2 of the 16S rRNA gene was amplified using PCR; this was followed by next-generation sequencing (NGS) library creation. The raw sequence data were analyzed using QIIME 2 (version 2023.5). Details are included in Supplementary Methods S1, Supplementary Methods S2, and Supplementary Table S1.

### Statistical Analysis

Unless otherwise stated, the results are presented as numbers (%) or medians. Comparisons between groups were performed using the independent *t*-test. Enumeration data were analyzed using the *χ*2 test. Cut-off values for predicting pathological responses were determined from receiver operating characteristic (ROC) curves of alpha diversity of the oral microbiome. Survival outcomes were evaluated in 62 consecutive surgical patients who were followed up for at least 2 years using the Kaplan–Meier method and compared using log-rank tests. Recurrence-free survival (RFS) was defined as the interval between the date of surgery and the first event (recurrence or death from any cause) or the most recent follow-up. Overall survival (OS) was defined as the time from the date of surgery until death owing to any cause or the last follow-up visit, whichever occurred first. Multivariate logistic regression analysis was performed to identify independent predictors of good pathological response. Multivariate Cox regression analysis was conducted to identify independent predictors of RFS. The backward stepwise method was used to select variables for multivariate analysis. Statistical analyses were performed using JMP Pro 18 software (SAS Institute, Cary, NC, USA, 2024). For comparisons between more than two groups, the Kruskal–Wallis test was performed, and for pairwise comparisons, the Mann–Whitney *U* test was used. *p* < 0.05 was considered significant.

### Microbial Analysis

To assess possible batch effects between sequencing runs (NGS11 and NGS12), permutational multivariate analysis of variance was conducted on unweighted UniFrac distance matrices. The analysis revealed a significant difference between runs (*p* = 0.001), indicating a batch effect that could influence downstream microbial community comparisons. To reduce this effect, a stratified random sampling approach was adopted. A total of 24 samples were chosen, six from four subgroups defined by pathological response (good versus poor responder) and sequencing run (NGS11 versus NGS12). Only samples that met the quality filtering criteria were available for selection. Random sampling was conducted with a fixed random seed (set.seed(42)) to ensure reproducibility. This sub-setting strategy was used to mitigate the effect of batch on downstream analysis, particularly in linear discriminant analysis effect size (LEfSe)-based differential abundance testing. LEfSe analysis was performed to identify differences in bacterial communities between the good and poor responder groups. When multiple identical taxa were detected, the taxon with the highest linear discriminant analysis (LDA) score was selected as a representative to identify the most biologically relevant features.^22^

## Results

### Clinicopathological Response to Neoadjuvant Therapy

The clinicopathological features of the 74 patients enrolled in this study are summarized in Table 1. In the RECIST evaluation of clinical response, 8 (10.8%) patients had a complete response and 47 (63.5%) had a partial response; the objective response rate for neoadjuvant therapy was 74.3%. A total of 18 (24.3%) patients had stable disease and 1 (1.4%) had progressive disease.

**Table 1:** Clinicopathological characteristics of the patients

Parameters	*n* = 74
Age (mean ± SD, years)	65.3 ± 11.3
*Sex*	
Male	58 (78.4%)
Female	16 (21.6%)
*Performance status*^a^	
0	55 (74.3%)
1	19 (25.7%)
*History of smoking*	
Never	13 (17.6%)
Former	43 (58.1%)
Current	18 (24.3%)
*Tumor markers*	
SCC (mean ± SD, ng/mL)	1.5 ± 1.4
CEA (mean ± SD, ng/mL)	4.4 ± 4.3
*Primary tumor location*	
Upper	15 (20.3%)
Middle	28 (37.8%)
Lower, Abdominal	31 (41.9%)
*Histology (biopsy specimens)*	
Well differentiated	6 (8.1%)
Moderately differentiated	18 (24.3%)
Poorly differentiated	9 (12.2%)
Squamous cell carcinoma (not assessable)	41 (55.4%)
*cT*^b^	
cT1	5 (6.7%)
cT2	11 (14.9%)
cT3	51 (68.9%)
cT4	7 (9.5%)
*cN*^b^	
cN0	20 (27.0%)
cN1	35 (47.3%)
cN2	18 (24.3%)
cN3	1 (1.4%)
*cM*^b^* (supraclavicular lymph node metastasis)*	
cM0	69 (93.2%)
cM1	5 (6.8%)
*Neoadjuvant therapy*	
Chemotherapy	65 (87.8%)
Chemoradiotherapy	9 (12.2%)
*Clinical response*^c^	
Complete response	8 (10.8%)
Partial response	47 (63.5%)
Stable disease	18 (24.3%)
Progressive disease	1 (1.4%)
*Histology (surgical specimens)*	
Well differentiated	5 (6.8%)
Moderately differentiated	20 (27.0%)
Poorly differentiated	18 (24.3%)
Squamous cell carcinoma (not assessable)	22 (29.7%)
No residual	9 (12.2%)
*Lymphatic invasion*	
0	64 (86.5%)
1	10 (13.5%)
*Venous invasion*	
0	56 (75.7%)
1	14 (18.9%)
2	4 (5.4%)
*pT*^d^	
pT0	9 (12.2%)
pT1	27 (36.5%)
pT2	11 (14.8%)
pT3	27 (36.5%)
*pN*^d^	
pN0	41 (55.4%)
pN1	15 (20.3%)
pN2	8 (10.8%)
pN3	10 (13.5%)
*pM*^d^* (supraclavicular lymph node metastasis)*	
pM0	71 (95.9%)
pM1	3 (4.1%)
*Pathological response*^e^	
Grade 0	6 (8.1%)
Grade 1	33 (44.6%)
Grade 2	26 (35.1%)
Grade 3	9 (12.2%)
*Alpha diversity of the oral microbiome*	
Shannon index (mean ± SD)	4.3 ± 0.63
Faith’s PD (mean ± SD)	6.2 ± 1.7
Observed features (mean ± SD)	44.0 ± 25.1

SCC, squamous cell carcinoma-related antigen CEA, carcinoembryonic antigen

^a^According to the Eastern Cooperative Oncology Group

^b^Clinical staging according to the tumor-node-metastasis classification, 8th edition

^c^According to the Response Evaluation Criteria in Solid Tumors

^d^Pathological staging according to the tumor-node-metastasis classification, 8th edition

^e^According to the Japan Esophageal Society of histopathological findings, 12th edition

In accordance with criteria recommended by the Japanese Society for Esophageal Diseases, individuals with grade 2 and 3 pathological responses were defined as good responders and those with grade 0 and 1 pathological responses were defined as poor responders.^22^ In total, 35 (47.3%) patients were good responders [grade 3, 9 (12.2%) patients; grade 2, 26 (35.1%) patients] and 39 (52.7%) were poor responders [grade 1, 33 (44.6%) patients; grade 0, 6 (8.1%) patients].

### Recurrence-Free and Overall Survival According to Pathological Response

**Fig 1:**
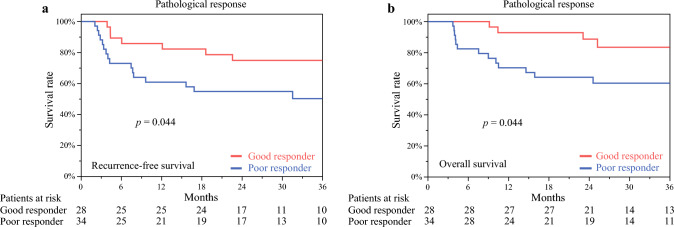
Survival analysis based on pathological response. **a** Recurrence-free and **b** overall survival stratified by pathological response. Responses were graded according to the Japanese Society for Esophageal Diseases guidelines (12^th^ edition); grades 2 and 3 were defined as good responders and grades 0 and 1 as poor responders

RFS based on pathological response is presented in Fig. 1a. The 1-, 2-, and 3-year RFS rates for the good and poor responders were 85.7% verus 60.7%, 74.8% versus 54.7%, and 74.8% versus 50.5%, respectively. Good responders exhibited significantly improved RFS than poor responders (*p* = 0.044). The OS according to pathological response is presented in Fig. 1b. The 1-, 2-, and 3-year OS rates for good and poor responders were 92.8% and 67.6%; 88.6% and 61.7%; and 83.4% and 58.5%, respectively. Good responders exhibited significantly improved OS than poor responders (*p* = 0.034).

### Association between Pathological Response and Oral Microbiome Diversity

**Fig. 2:**
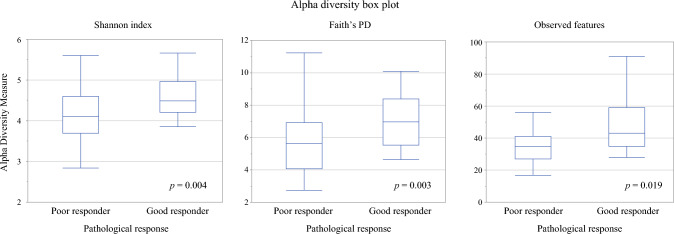
Association between pathological response and alpha diversity in the oral microbiome. Association between pathological response and alpha diversity in the oral microbiome. Alpha diversity was evaluated using the Shannon index, Faith’s PD, and observed features. Responses were graded according to the Japanese Society for Esophageal Diseases guidelines (12^th^ edition); grades 2 and 3 were defined as good responders, and grades 0 and 1 were defined as poor responders. Box plots represent medians, first and third quartiles, and the minimum and maximum values

The relationship between pathological response and alpha diversity of the oral microbiota is presented in Fig. 2. Alpha diversity (Shannon index, Faith’s PD, and observed features) correlated with pathological response and was significantly higher in good responders (Shannon index, *p* = 0.004; Faith’s PD, *p* = 0.003; observed features, *p* = 0.019). Using ROC curve analysis, the optimal cutoff values identified for predicting a good treatment response were 4.1 for the Shannon index (area under the curve [AUC] 0.70; 95% confidence interval [CI], 0.55–0.79; *p* = 0.0004), 5.9 for Faith’s PD (AUC 0.70; 95% CI, 0.56–0.79; *p* = 0.0007), and 32.0 for observed features (AUC 0.73; 95% CI, 0.59–0.82; *p* = 0.0003). The relationships between the cut-off values of oral microbiota alpha diversity (Shannon index, Faith PD’s, and observed features) and treatment efficacy are presented in Supplementary Table S2. Multivariate logistic regression and Cox regression analyses were conducted to avoid confounding factors using the observed features with the highest AUC values among the three alpha diversity indices.

### Univariate and Multivariate Analyses for Good Pathological Response

The results of the univariate and multivariate analyses of the predictors of a good pathological response are presented in Table 2. Univariate analysis showed that the alpha diversity of the oral microbiome (observed features, high > 32, low < 32; high versus low; odds ratio [OR]: 9.14, 95% confidence interval [CI]: 2.68–42.56, *p* = 0.0002) was significantly correlated with the pathological response. Multivariate analyses indicated that the alpha diversity of the oral microbiome (observed features, high > 32, low < 32; high versus low; OR: 11.46, 95% CI: 2.49–52.64, *p* = 0.002) was significantly correlated with the pathological response.

**Table 2:** Results of univariate and multivariate analyses of factors associated with a good pathological response

Variables	Univariate			Multivariate		
	OR	95% CI	*p* value	OR	95% CI	*p* value
Age, years (continuous)	0.97	0.92–1.01	0.217	–	–	–
*Sex*						
Female (reference)	1			–	–	–
Male	0.87	0.28–2.67	0.806	–	–	–
*Performance status*^a^						
0 (reference)	1			–	–	–
1	0.75	0.25–2.14	0.598	–	–	–
SCC (continuous)	1.02	0.72–1.42	0.898	–	–	–
CEA (continuous)	0.97	0.86–1.09	0.702	–	–	–
*Primary tumor location*						
Middle, Lower (reference)	1			–	–	–
Upper	0.68	0.20–2.15	0.524	–	–	–
*Histology (biopsy specimens)*						
Not poorly differentiated (reference)	1			–	–	–
Poorly differentiated	0.41	0.05–2.05	0.288	–	–	–
*cT*^b^						
1/2 (reference)	1			–	–	–
3/4	0.45	0.13–1.39	0.167	–	–	–
*cN*^b^						
0/1 (reference)	1			–	–	–
2/3	0.75	0.25–2.14	0.598	–	–	–
*cM*^b^ (*supraclavicular lymph node metastasis*)						
0 (reference)	1			–	–	–
1	0.72	0.09–4.64	0.734	–	–	–
*Neoadjuvant therapy*						
Chemotherapy (reference)	1			–	–	–
Chemoradiotherapy	1.70	0.48–6.30	0.403	–	–	–
*Alpha diversity of oral microbiome (observed features)*^c^						
Low (reference)	1			1		
High	9.14	2.68–42.56	0.0002	11.46	2.49–52.64	0.002

OR, odds ratio, CI, confidence interval, SCC, squamous cell carcinoma-related antigen, CEA, carcinoembryonic antigen

^a^According to the Eastern Cooperative Oncology Group

^b^Clinical staging according to the tumor-node-metastasis classification, 8th edition

^c^Observed features, high > 32, low < 32

### Univariate and Multivariate Analyses for Recurrence-Free Survival

The results of univariate and multivariate analyses of prognostic factors for RFS are presented in Table 3. Univariate analysis showed that the histology (resected specimens; poorly differentiated versus not poorly differentiated; hazard ratio [HR]: 2.53, 95% CI: 1.04–6.18, *p* = 0.041), pathological T factor (2/3 versus 0/1; HR: 8.13, 95% CI: 3.52–18.74, *p* <0.0001), pathological N factor (2/3 versus 0/1; HR: 3.52, 95% CI: 1.66–7.49, *p* = 0.001), pathological response (Grade 0/1 versus Grade 2/3; HR: 2.45, 95% CI: 1.02–5.81, *p* = 0.044), and alpha diversity of the oral microbiome (observed features, high > 32, low < 32; low versus high; HR: 2.77, 95% CI: 1.21–6.36, *p* = 0.015) were significantly correlated with RFS. Multivariate analyses revealed that the pathological T factor (2/3 versus 0/1; HR: 5.59, 95% CI: 2.03–15.37, *p* = 0.001), pathological N factor (2/3 versus 0/1; HR: 4.09, 95% CI: 1.58–10.55, *p* = 0.003), pathological response (Grade 0/1 versus Grade 2/3; HR: 2.46, 95% CI: 1.01–6.01, *p* = 0.048), and alpha diversity of the oral microbiome (observed features, high > 32, low < 32; low versus high; HR: 3.89, 95% CI: 1.57–9.65, *p* = 0.005) were significantly correlated with RFS.

**Table 3:** Results of univariate and multivariate analyses of prognostic factors for recurrence-free survival

Variables	Univariate			Multivariate		
	HR	95% CI	*p* value	HR	95% CI	*p* value
Age, years (continuous)	1.01	0.96–1.06	0.746	–	–	–
*Sex*						
Female (reference)	1			–	–	–
Male	1.17	0.43–3.17	0.748	–	–	–
*Performance status*^a^						
0 (reference)	1			–	–	–
1	1.71	0.70–4.17	0.237	–	–	–
SCC (continuous)	1.02	0.72–1.42	0.898	–	–	–
CEA (continuous)	1.02	0.93–1.08	0.484	–	–	–
*Primary tumor location*						
Middle, Lower (reference)	1			–	–	–
Upper	1.34	0.52–3.40	0.534	–	–	–
*Neoadjuvant therapy*						
Chemotherapy	1			–	–	–
Chemoradiotherapy	2.39	0.93–6.12	0.067	–	–	–
*Histology (resected specimens)*						
Not poorly differentiated (reference)	1			–	–	–
Poorly differentiated	2.53	1.04–6.18	0.041	–	–	–
*pT*^b^						
0/1 (reference)	1			1		
2/3	8.13	3.52–18.74	<0.0001	5.59	2.03–15.37	0.001
*pN*^b^						
0/1 (reference)	1			1		
2/3	3.52	1.66–7.49	0.001	4.09	1.58–10.55	0.003
*pM*^b^* (supraclavicular lymph node metastasis)*						
0 (reference)	1			–	–	–
1	2.49	0.59–10.54	0.214	–	–	–
*Pathological response*^c^						
Grade 2/3 (reference)	1			1		
Grade 0/1	2.45	1.02–5.81	0.044	2.46	1.01–6.01	0.048
*Alpha diversity of oral microbiome* (*observed features*)^d^						
High (reference)	1			1		
Low	2.77	1.21–6.36	0.015	3.89	1.57–9.65	0.005

HR, hazard ratio, CI*,* confidence interval, SCC, squamous cell carcinoma-related antigen, CEA, carcinoembryonic antigen

^a^According to the Eastern Cooperative Oncology Group

^b^Pathological staging according to the tumor-node-metastasis classification, 8th edition

^c^According to the Japanese Classification of Esophageal Cancer, 12th Edition (Japan Esophageal Society)

^d^Observed features, high > 32, low < 32

### Recurrence-Free Survival and Overall Survival According to Alpha Diversity of the Oral Microbiome

**Fig. 3:**
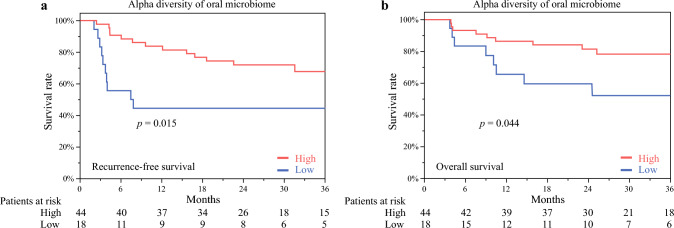
Survival rate according to the alpha diversity in the oral microbiome**. a** Recurrence-free and **b** overall survival according to alpha diversity of the oral microbiome (observed species, high > 32, low < 32)

The RFS, according to the alpha diversity of the oral microbiome (observed species: high > 32, low < 32), is presented in Fig. 3a. The 1-, 2-, and 3-year RFS rates for high- and low-alpha diversity cases were 83.7% versus 44.0%, 71.9% versus 44.0%, and 67.9% versus 44.0%, respectively. Patients with high alpha diversity showed significantly improved RFS than those with low alpha diversity (*p* = 0.015).

The OS, as indicated by the alpha diversity of the oral microbiome (observed species: high > 32, low < 32), is presented in Fig. 3B. The 1-, 2-, and 3-year OS rates for high- and low-alpha diversity cases were 86.3% versus 61.1%, 81.4% versus 55.5%, and 78.4% versus 49.3%, respectively. Patients with high-alpha diversity had significantly improved OS than those with low alpha-diversity (*p* = 0.044).

### Oral Microbiome Characteristics According to Pathological Response

**Fig. 4:**
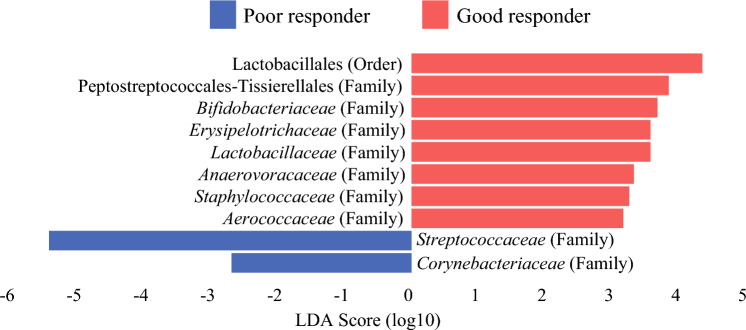
Characteristics of the oral microbiome according to pathological responses. Characteristics of the oral microbiome according to pathological responses. Differences in the relative abundance of bacterial communities between the two groups based on linear discriminant analysis (LDA) effect size and p-value (LDA > 2.0 and *p* < 0.05). Responses were graded according to the Japanese Society for Esophageal Diseases guidelines (12^th^ edition); individuals with grade 2 and 3 responses were defined as good responders, and those with grade 0 and 1 responses were defined as poor responders

The characteristics of the oral microbiome according to pathological response are presented in Fig. 4. LEfSe analysis was performed on a stratified subset of 24 high-quality, nonexcluded samples (six from each subgroup: good/poor × NGS11/NGS12) to minimize batch effects between sequencing runs. LEfSe analysis was performed to identify bacterial communities that differed between the good- and poor-responder groups. The highest LDA score was selected as a representative to identify the most biologically relevant features when multiple identical taxa were detected. In total, 10 bacterial groups met the LDA score criteria of > 2.0 and had a between-group *p*-value of < 0.05. Eight bacterial groups (*Lactobacillales*, *Peptostreptococcales-Tissierellales*, *Bifidobacteriaceae*, *Erysipelotrichaceae*, *Lactobacillaceae*, *Anaerovoracaceae*, *Staphylococcaceae*, and *Aerococcaceae*) were significantly more abundant in the good-responder group, and two bacterial groups (*Streptococcaceae* and *Corynebacteriaceae*) were significantly more abundant in the poor-responder group.

## Discussion

In this study, we demonstrated the association between the oral microbiome and treatment efficacy and prognosis using oral microbiome metagenomic data obtained from patients with ESCC. Furthermore, the characteristic bacterial populations of good and poor responders were identified. To the best of our knowledge, this is the first study to examine the relationship between the metagenomic data of the oral microbiota and treatment efficacy.

Research on the gut microbiota has yielded a wide range of findings, from carcinogenesis to the mechanisms influencing the effectiveness of chemotherapy and immune checkpoint inhibitors.^6–10,23–25^ The oral microbiota plays a crucial role in periodontal disease and is essential in maintaining human health. Furthermore, it contributes to the establishment of the microbiota in the digestive tract.^11,12,26,27^ Similar to the gut microbiota, the oral microbiota is reportedly involved in carcinogenesis, and many studies have shown an association with oral and head and neck cancers, especially. Recently, there have also been suggestions regarding its association with esophageal cancer.^13–15,28^

With advances in metagenomic analyses, a growing body of research has emerged on the correlation between bacterial metagenomics and cancer. Differences in the composition of the microbiome were observed between colorectal cancer and healthy colorectal tissues, with α diversity being lower in the cancer tissues compared with the normal tissues. The *α* diversity of intestinal bacteria is also lower in colorectal adenomas, indicating a potential association between the carcinogenic pathway and intestinal bacterial diversity.^29^ In pancreatic cancer, the tumor microbiome of patients with long-term survival has high alpha diversity, and a tumor microbiome signature that predicts long-term survival rates has been identified.^30^

Several studies have shown the correlation between bacterial diversity and drug therapies. The gut microbiome composition of patients with melanoma who responded to immune checkpoint inhibitor therapy differed from those who did not respond, and alpha diversity was higher.^31^ Furthermore, in patients with non-small cell lung cancer treated with immune checkpoint inhibitors, the intestinal microbiota of those who responded to treatment exhibited high alpha diversity, a stable bacterial composition, and an extended progression-free survival period.^32^

Metagenomic data on the gut microbiota in relation to esophageal cancer are relatively scarce compared with other cancers; however, several noteworthy studies have been reported. In a study comparing the gut microbiota of patients with esophageal cancer and healthy individuals, in the gut microbiome of patients with ESCC, an increase was noted in potentially inflammatory and/or carcinogenic bacteria, and a decrease in butyrate-producing and/or potentially antiinflammatory bacteria, such as *Butyricicoccus*, *Lachnospiraceae NK4A136 group*, and *Eubacterium eligens group*.^33^

Reports on the relationship between oral microbiota metagenomic data and esophageal cancer are more limited than reports on gut microbiota. Certain studies have identified bacteria such as *Tannerella forsythia*, *Streptococcus anginosus*, *Aggregatibacter actinomycetemcomitans*, *Treponema denticola*, and *Streptococcus mitis* as potential risk factors for esophageal cancer based on metagenomic data of oral bacteria in esophageal cancer patients.^29,34^ To date, no reports examining the association between metagenomic data of oral microbiota and treatment efficacy in esophageal cancer have been published, making this study the first of its kind.

In addition, this study demonstrated the bacterial community characteristics of the pathological response groups with good and poor outcomes. Further research is needed to investigate the mechanisms that influence treatment efficacy, but for example, *Bifidobacterium* are abundant in good responders, aiding in promoting tumor immunity. This process is believed to be associated with the maturation of dendritic cells and the activation of CD8+ T cells. It also increases the production of IFNγ, IFNα, and interleukin (IL)-17, which improves the tumor microenvironment and promotes tumor immunity, thereby contributing to enhanced efficacy of drug therapy. The combination of anti-PD-L1 antibodies and *Bifidobacterium* enhances tumor shrinkage.^35,36^ Furthermore, it antagonizes the effects of the carcinogen 2-amino-3-methylimidazo [4,5-f] quinolone and protects DNA from damage induced by carcinogens, thereby reducing carcinogenic risk.^37^

*Streptococcus*, which is abundant in poor responders, induces tumor immune suppression. The process is believed to involve the inhibition of macrophage differentiation, thereby increasing IL-10 expression in tumor tissue and suppressing the function of immune cells, such as CD8+ T cells, dendritic cells, and NK cells.^38^ Reports have also shown that *Streptococcus* inhibits IL-17 signaling and reduces the number of CD8+IL17A+ tissue-resident memory T-cells.^39^ Administering *Bifidobacterium* as a probiotic to compete with *Streptococcus* increased IFN-γ expression and promoted tumor shrinkage.^38^ These findings link diversity to immunity. Low diversity favors suppressive *Streptococcus* spp., whereas high diversity preserves beneficial *Bifidobacterium* spp. that aid in the antitumor response.

Several biological mechanisms may further explain how the oral microbiota influences treatment efficacy. Recent studies have shown that oral bacteria can modulate systemic immunity by circulating microbial metabolites, such as short-chain fatty acids, polyamines, and bacterial outer membrane vesicles. These metabolites can promote regulatory T-cell differentiation, suppress cytotoxic T-cell function, and activate pro-survival pathways in tumor cells.^40^ Oral species, such as *Fusobacterium nucleatum,* can also translocate to esophageal and gastrointestinal tumors via micro-aspiration or transient bacteremia, where they enhance autophagy and chemoresistance.^41^

Thus, manipulation of oral microbiota holds promise as a future treatment alternative. Stabilization of the oral microbiota using antibiotics, prebiotics, probiotics, and microbiota transplantation may improve the efficacy of esophageal cancer treatment.^38,42^ Dental treatment may shift the microbial profile toward a less pathological state. Furthermore, combining conventional dental treatment with bacterial treatments, such as *Bifidobacterium lactis,* could increase the abundance of health-associated bacteria.^43^ Although direct evidence in esophageal cancer is lacking, probiotics can boost antitumor immunity in melanoma.^35^ Because oral bacteria directly reach the esophagus, similar mechanisms may apply. Future studies are required to investigate whether combining dental interventions with microbial strategies will influence treatment response in esophageal cancer.

This study has certain limitations. This was a single-center study; therefore, limitations occurred because of the sample size. Further research is required to elucidate the mechanism by which the oral microbiota identified in this study affects the efficacy of esophageal cancer therapeutics and to determine whether intervention in the oral microbiota can improve therapeutic efficacy. To comprehensively evaluate both treatment efficacy and the impact of the oral microbiome on long-term outcomes, future studies should include patients who received only surgery.

In conclusion, this study showed that the alpha diversity of the oral microbiota correlates with the pathological response to neoadjuvant therapy for ESCC and reflects prognosis. In addition, characteristic bacterial populations were identified in the groups with both good and poor treatment responses. These findings may contribute not only to predicting the therapeutic efficacy and prognosis of esophageal cancer treatment, but also to enhancing its effectiveness.

## Supplementary Information

Below is the link to the electronic supplementary material.

Supplementary file1 (DOCX 30 KB)

## Data Availability

The sequence data are available in the DDBJ Sequence Read Archive under BioProject number PRJDB35618 (Run accession numbers: DRR706031–DRR706113).
