# Supplementary material for: Relationship Between the Oral Microbiome and Treatment Efficacy in Esophageal Squamous Cell Carcinoma
Source: Ann Surg Oncol. 2026 Jan 12;33(4):3203–13. doi: 10.1245/s10434-025-18945-8 (PMC12982235; doi:10.1245/s10434-025-18945-8)
Supplement: Supplementary file 1 — Supplementary file1 (DOCX 30 KB) [file 10434_2025_18945_MOESM1_ESM.docx]

**Relationship between the oral microbiome and treatment efficacy in esophageal squamous cell carcinoma**

Manato Ohsawa^1^, Hiromi Nishi^2^, Yoichi Hamai^1^, Manabu Emi^1^, Yuta Ibuki^1^, Hitoshi Komatsuzawa^3^, Hiroyuki Kawaguchi^2^, Morihito Okada^1^

^1^Department of Surgical Oncology, Hiroshima University, Hiroshima, Japan

^2^Department of General Dentistry, Hiroshima University Hospital, Hiroshima, Japan

^3^Department of Bacteriology, Hiroshima University Graduate School of Biomedical and Health Sciences, Hiroshima, Japan

**Corresponding author.** Hiromi Nishi
Department of General Dentistry, Hiroshima University, 1-2-3-Kasumi, Minami-ku, Hiroshima City, Hiroshima 734-0037, Japan

**Supplementary Materials - Index**

| **Supplementary Methods** | | |  | |
| --- | --- | --- | --- | --- |
| Supplementary Methods S1. DNA extraction, 16S rRNA library, and NGS library production | | | *page 2* | |
| Supplementary Methods S2. Sequence analysis | | | *page 3* | |
| **Supplementary Figures and Tables** |  | |  |  |
| Table S1 Sample exclusion summary based on DADA2 filtering | *page 4* | |  |  |
| Table S2. Prediction of treatment efficacy using alpha diversity of the oral microbiota | *page 5* | |  |  |

**Supplementary methods**

**Supplementary Methods S1. DNA extraction, 16S rRNA library, and NGS library production**

The obtained samples were suspended in 1 mL phosphate-buffered saline and immediately stored at −80 °C until further use. DNA was extracted using a MasterPure Complete DNA/RNA Purification Kit (Epicenter, Madison, WI, USA) according to the manufacturer’s instructions and then dissolved in 20 μL EB buffer. Polymerase chain reaction (PCR) was performed once using a set of Illumina index primers to amplify the hypervariable V1-V2 region of the 16S rRNA gene and for indexing (Illumina, San Diego, CA, USA). The total volume of reaction liquid was 25 μL, comprising 1 μL used for the examination, DNA template, 12.5 μL KAPA HiFi HotStart ReadyMix (KAPA Biosystems, Wilmington, MA, USA), and primers for indexing. The PCR process was as follows: initial denaturation at 95 °C for 2 min, followed by 30 cycles at 95 °C for 30 s, 55 °C for 30 s, and 72 °C for 30 s, with a final extension at 72 °C for 5 min. PCR products were purified using AMPure XP magnetic beads (Beckman Coulter, Brea, CA, USA), followed by elution with EB buffer. The DNA concentration was then determined using a Nanodrop device. Each sample was mixed at 500 ng, and DNA was purified using a QIAquick PCR Purification Kit (Qiagen, Hilden, Germany) according to the manufacturer’s instructions. The resulting sample was used as a library for NGS.

The density of the library was determined using a Qubit fluorometer (Thermo Fisher Scientific, Inc., Waltham, MA, USA) and adjusted to a final concentration of 5 pM. A 40% phiX control was added to enhance sequence diversity. Libraries were sequenced using an Illumina MiSeq system with 2 × 300 bp paired-end reads and the MiSeq Reagent Kit v3 (600 cycles), with data acquired in FASTQ format. The sequence data are available in the DDBJ Sequence Read Archive under BioProject number PRJDB35618 (Run accession numbers: DRR706031–DRR706113).

**Supplementary Methods S2. Sequence analysis**

Denoising and chimera removal were performed before using the DADA2 plugin to create amplicon sequence variants. According to the quality profiles, forward and reverse reads were trimmed at 270 and 200 bp ends, respectively. Nine low-quality samples were excluded based on quality criteria and 74 samples were used in the final analysis. The sequence data from two sequencing runs (NGS11 and NGS12) were analyzed. Samples from NGS12 met the quality criteria and were retained. In comparison, nine low-quality samples from NGS11 were discarded using less stringent quality cut-off values. A total of 83 samples were initially processed. The exclusion criteria and sample IDs are provided in Table S1.

Before the diversity and statistical analyses, feature tables from both sequencing runs were combined. Rarefaction of each sample was performed at a depth of 4,500 reads to standardize the data for downstream analyses. Taxonomic classification was performed using a custom-trained Naive Bayes classifier generated using the q2-feature-classifier plugin in QIIME 2. The classifier was trained on the SILVA 138 reference database and trimmed to match the V1–V2 region of the 16S rRNA gene (primers 27Fmod–338R), which corresponds to the amplicon region used in this study. Taxonomic assignments were performed at a 97% sequence similarity.

| **Table S1.** **Sample exclusion summary based on DADA2 filtering.** | | | |
| --- | --- | --- | --- |
| **Sequencing run** | **Sample IDs used for analysis** | **Excluded sample IDs** | **Exclusion criteria** |
| NGS11 | 4, 5, 6, 12, 15, 18, 19, 20, 22, 24, 25, 27, 28, 29, 30, 31, 32, 34, 37, 38, 39, 42, 43, 49, 51, 52, 54, 58, 61, 64, 71, 77, 82, 86, 87, 89, 91, 94, 102, 103, 105, 107, 108, 109, 111, 113, 117, 120, 122, 123, 125, 130, 132, 133, 134, 136, 138, 145, 148 | 11, 26, 44, 68, 72, 73, 79, 81, 93 | Samples were excluded if any of the following thresholds were met:  (i) Non-chimeric reads < 7% (ii) Reads retained after filtering < 8% (iii) Merged read success rate < 6.5%  (iv) Non-chimeric read count < 4,500 |
| NGS12 | 47, 100, 139, 140, 141, 142, 143, 146, 147, 149, 150, 160, 162, 196 | None | Not applicable |

| **Table S2.** **Prediction of treatment efficacy using alpha diversity of the oral microbiota.** | | |  |
| --- | --- | --- | --- |
|  | Good responder^a^ (grade 2 or 3) | Poor responder^a^ (grade 0 or 1) | p-value |
|  | n = 35 | n = 39 |  |
| Alpha diversity (Shannon index) |  |  |  |
| Low (< 4.1) | 5 (19.2%) | 21 (80.8%) | 0.0004 |
| High (> 4.1) | 30 (62.5%) | 18 (37.5%) |  |
| Alpha diversity (Faith PD) |  |  |  |
| Low (< 5.9) | 11 (31.4%) | 24 (68.6%) | 0.009 |
| High (> 5.9) | 24 (61.5%) | 15 (38.5%) |  |
| Alpha diversity (observed features) |  |  |  |
| Low (< 32) | 3 (14.3%) | 18 (85.7%) | 0.0003 |
| High (> 32) | 32 (60.4%) | 21 (39.6%) |  |
| PD, progressive disease  ^a^ Responses were graded according to the Japanese Society for Esophageal Diseases guidelines (12^th^ edition); grades 2 and 3 were defined as good responders, and grades 0 and 1 were defined as poor responders. | | | |
